# Comprehensive Improvement of the Sensitivity and Detectability of a Large-Aperture Electromagnetic Wear Particle Detector

**DOI:** 10.3390/s19143162

**Published:** 2019-07-18

**Authors:** Ran Jia, Biao Ma, Changsong Zheng, Xin Ba, Liyong Wang, Qiu Du, Kai Wang

**Affiliations:** 1 School of Mechanical Engineering, Beijing Institute of Technology, Zhongguancun South Street No.5 Haidian District, Beijing 100081, China; 2 Faculty of Engineering and Information, University of Technology Sydney, Ultimo, NSW 2007, Australia; 3 The Ministry of Education Key Laboratory of Modern Measurement and Control Technology, Beijing Information Science and Technology University, Xiaoying East Street No.12, Beijing 100192, China

**Keywords:** particle detection, sensitivity, resonance, amorphous core, signal extraction

## Abstract

The electromagnetic wear particle detector has been widely studied due to its prospective applications in various fields. In order to meet the requirements of the high-precision wear particle detector, a comprehensive method of improving the sensitivity and detectability of the sensor is proposed. Based on the nature of the sensor, parallel resonant exciting coils are used to increase the impedance change of the exciting circuit caused by particles, and the serial resonant topology structure and an amorphous core are applied to the inductive coil, which improves the magnetic flux change of the inductive coil and enlarges the induced electromotive force of the sensor. Moreover, the influences of the resonance frequency on the sensitivity and effective particle detection range of the sensor are studied, which forms the basis for optimizing the frequency of the magnetic field within the sensor. For further improving the detectability of micro-particles and the real-time monitoring ability of the sensor, a simple and quick extraction method for the particle signal, based on a modified lock-in amplifier and empirical mode decomposition and reverse reconstruction (EMD-RRC), is proposed, which can effectively extract the particle signal from the raw signal with low signal-to-noise ratio (SNR). The simulation and experimental results show that the proposed methods improve the sensitivity of the sensor by more than six times.

## 1. Introduction

Wear is one of the major causes of failure in machine components. The excessive wear of some core parts of machineries, especially for large-scale mechanical equipment, may lead to a poor mechanical performance, which in turn causes enormous economic losses. Therefore, for online monitoring of the wear condition of machineries in order to prevent serious malfunctions, the wear particle detector has demonstrated its value [1,2,3]. To date, wear particle detectors with different physical principles, including optics, ultrasonics, electronics, and imaging, have been proposed, and the characteristics of the various kinds of sensors are listed in Reference [4]. Among them, electromagnetic wear particle detectors have demonstrated significant advantages in online wear condition monitoring because of their strong anti-interference ability, good temperature stability, and high reliability. 

To achieve a better particle detection effect, sensors with different structures have long been objects of study. Flanagan et al. [5] proposed a wear particle detector with a single coil (inner diameter of 6 mm), which identifies particles by the fluctuation of the sensor resonance frequency. Experimental results showed that the sensor could detect iron particles with a diameter of 150 μm. Fan et al. [6] designed a double-coil wear particle detection sensor. It estimates the size and the material properties of particles by measuring the inductance difference between the sensing coil and the reference coil of the sensor and can successfully detect 100 μm ferromagnetic particles and 500 μm non-ferromagnetic particles. To improve the consistency of the particle detection results, a sensor with planar spiral coils [7] was proposed. The simulation and experimental results showed that the uniformity of the magnetic field in the detection area was greatly improved, however, the sensor could only detect the ferromagnetic particles with a diameter of 700 μm. Further, Hong et al. [8] designed a radial inductive debris detection sensor that consisted of a C-type iron core, a drive coil, and an inductive coil. The experimental results indicated that the sensor could effectively detect a 290 μm ferromagnetic particle in a 20 mm diameter pipe. However, the magnetostatic field was adopted in this sensor, so it could not detect non-ferromagnetic particles. To improve the sensitivity of the sensor, the wear particle detector with a parallel three-coil structure was studied [9,10,11]. The study demonstrated that the sensor could detect approximately 100 μm ferromagnetic particles and 305 μm non-ferromagnetic particles in a 7.6 mm diameter channel. However, the sensitivity and the detectability are still the main obstacles for the development and application of the wear particle detector. Therefore, some measures have been taken to further improve the sensitivity of the sensor. The most direct and valid approach is adopting micro-channel structures [12]. The typical feature of this kind of sensor is that the diameter of the inner channel is smaller than 1 mm, which reduces the distance between target particles and sensor coils. Du et al. [13] proposed a micro-channel device based on an inductive coulter counting principle to detect metal wear particles in lubricating oil. The device could detect about 50 μm ferromagnetic particles and 125 μm non-ferromagnetic particles. Wu et al. [14] designed a microfluidic chip-based inductive wear particle detection device. For this sensor, the inner diameter of the coil was set to 200 μm, and the experimental results revealed that it could detect ferromagnetic particles with a diameter of 5–10 μm. Although the sensitivity of the sensor was greatly enhanced, the small channel diameter of the sensor greatly limits its application to large-scale machineries. Besides that, Li et al. [15] carried out a study to improve the sensitivity of a single-coil wear particle detector. They innovatively proposed that adding an external capacitor to the sensor coil and making the sensor work in a parallel resonance state could boost the sensitivity of the sensor. Recently, Zhu et al. [16] added a ferrite core to the single-coil wear debris detection sensor for the enhancement of sensor sensitivity. With this method, the sensor could detect 11 μm ferromagnetic particles in fluidic pipes with a diameter of 1 mm under a throughput of 750 mL/min.

The size of the minimum detectable particle and the real-time ability of the sensor are also limited by the noise level of the raw signal and the performance of the particle signal extraction algorithm. Fan et al. [17] presented a joint time-invariant wavelet transform and kurtosis analysis approach to extract the effective particle signal. This method depresses the background noise of a raw signal by a threshold. In this way, the wear particle detection effect is greatly influenced by the environmental noise. Li et al. [10,18,19] adopted the maximal overlap discrete wavelet transform to remove vibration interferences from the raw signal. Luo et al. [20] integrated the resonance-based signal decomposition method and fractional calculus (RSD-FC) to improve the detection accuracy of the sensor. These methods do improve the particle detection effect to a certain degree, but they are only valid when the signal-to-noise ratio (SNR) of the signal is sufficiently high, which generally means higher than 2 dB. Meanwhile, overcomplicated algorithms require a relatively high computational cost, which makes the sensor unsuitable for application to continuous real-time monitoring [21].

To meet the requirements of the high-precision wear particle detector and improve the micro-particle detection effect, a comprehensive method of improving the sensitivity and detectability of the sensor is proposed. Based on the essential features of the sensor, a parallel resonance topology and a series resonance topology are applied to the exciting coil and the inductive coil respectively, to comprehensively boost the sensitivity of the sensor. In addition, the influence of resonance frequency on the sensitivity and effective particle detection range of sensors is studied, which lays the foundation for optimizing the frequency of the magnetic field within the sensor. To further improve the induced electromotive force, an amorphous iron core is added to the inductive coil. The high permeability and the low hysteresis loss and eddy current loss of the amorphous material contribute to improving the sensitivity and keeping the performance of the sensor under a high-frequency alternating magnetic field. Additionally, to improve the real-time performance of wear monitoring, a quick extraction method of the particle signal, based on a modified lock-in amplifier and empirical mode decomposition, is proposed. This method dramatically reduces the amount of computation of the system and can quickly extract the particle signal from the raw signal with an extremely low signal-to-noise ratio (SNR).

## 2. Device Description and Measurement Setup

### 2.1. Sensor Description

**Figure 1:**
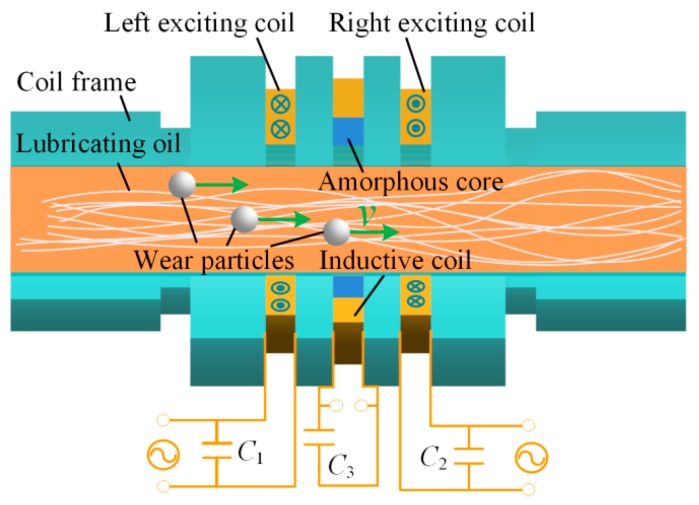
The structure of the proposed wear particle detector.

The core structure of the proposed wear particle detector is shown in Figure 1. Differing from the conventional wear particle detection sensor, which only includes a coil frame, two reverse exciting coils, and an inductive coil, the proposed particle detector adopts the resonance principle and an amorphous iron core to compressively improve its sensitivity. Based on the features of the sensor, the parallel resonance topology is used for the exciting coil to boost the impedance change of the coil caused by particles. Moreover, the series resonance principle is applied to the inductive coil to improve the induced electromotive force. Therefore, the resonant capacitors *C*_1_ and *C*_3_ are connected to the left and right exciting coils of the sensor in parallel, and the resonant capacitor *C*_2_ is connected to the inductive coil in series. The general working principle of the sensor has been expounded in Reference [22]. In order to achieve the flow requirements of wear monitoring for large-scale machines, the inner diameter of the sensor is set to 7 mm.

The metal wear particles passing through the sensor lead to magnetic perturbation of the sensor. More specifically, ferromagnetic particles enhance the local magnetic flux density, while non-ferromagnetic particles decrease the local magnetic flux density [22]. In these cases, the change of the magnetic flux through the exciting coil and the inductive coil can be expressed as (1) and (2), respectively:

(1) $$\Delta \phi_{e}=\sum \int \Delta B_{p}\left(x,y\right)ds=\Delta (L\times I)$$

(2) $$\Delta \phi_{i}=K(1-\lambda )(\phi_{e1}-\phi_{e2})$$

where, $\phi_{e}$ is the magnetic flux through the exciting coil, $\Delta B_{p}$ is the change of magnetic flux density in the sensor caused by particles, *L* is the inductance of the exciting coil, *I* is the current through the exciting coil, K is the gain factor of magnetic flux through the inductive coil, λ is the magnetic flux leakage coefficient, which is closely related to the sensor structural parameters, and $\phi_{ei}$ is the magnetic flux through the *i*th exciting coil.

The induced electromotive force output by the inductive coil can be expressed as (3), where $N_{i}$ is the number of turns of the inductive coil:

(3) $$E_{0}=-N_{i}\frac{\Delta \phi_{i}}{\Delta t}\approx -KN_{i}(1-\lambda )\frac{\Delta (L\times I)}{\Delta t}.$$

From the above equation, we can see that for the sensor with certain structural parameters, the magnitude of the induced electromotive force is related to the product of the inductance of the exciting coil and current through the exciting coil, and the gain factor K. Because the change of coil inductance caused by wear particles is extremely weak, one method of improving the sensitivity of the sensor is to enlarge the current variation through the exciting coils, which is closely associated with the impedance change of the exciting circuit caused by particles. Meanwhile, this research proves that a series-resonant inductive coil and an amorphous core can boost the gain factor K. The mechanism of enhancing the sensitivity of the sensor is explained in detail in the following section.

### 2.2. A Sensitivity Comparison Analysis of the Sensors

**Figure 2:**
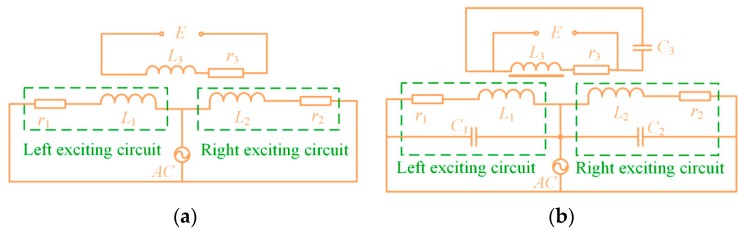
The circuit diagrams of the sensors. (**a**) The conventional sensor, (**b**) the proposed sensor.

To demonstrate the mechanism of sensitivity improvement by the resonant principle and the amorphous core, a sensitivity comparison analysis of the conventional and proposed wear particle detector was conducted. The circuit diagrams of the sensors are displayed in Figure 2a,b, where *L*_1_ and *L*_2_ are the inductances of the exciting coils, *L*_3_ is the inductance of the inductive coil, *C*_1_, *C*_2_, and *C*_3_ are the resonant capacitors for each coil, and the internal resistances of these coils are *r*_1_ = *r*_2_ = 4.1 Ω and *r*_3_ = 4.3 Ω. For the proposed sensor, as shown in Figure 2b, the resonance condition must be satisfied as Equation (4), where $f_{0}$ is the resonant frequency.

(4) $$f_{0}\approx \frac{1}{2\pi \sqrt{LC}}$$

The impedance change of the exciting circuit caused by particles can characterize the sensitivity of the sensor indirectly. When no particles enter the sensor, the impedance of each exciting circuit of the two sensors, as shown in Figure 2a,b, can be expressed as (5) and (6), respectively. Here, $Z_{a}$ and $Z_{b}$ are the impedances of the non-resonant and resonant exciting circuits respectively, $L_{q}=L_{i}-M$ is the equivalent inductance of a single exciting coil, $L_{i}$ is the self-inductance of the *i*th exciting coil, and *M* is the mutual inductance between the two exciting coils. Note that, under the resonance state, $1-\omega^{2}L_{q}C\approx 0$ and ωCr≪1, so it can be obtained that $Z_{b}\gg Z_{a}$.

(5) $$Z_{a}=j\omega L_{q}+r$$

(6) $$Z_{b}=\frac{\left(j\omega L_{q}+r\right)}{1-\omega^{2}L_{q}C+j\omega Cr}.$$

When wear debris gets access to the sensor, the inductance of one of the two exciting coils changes, which further leads to an impedance difference between the two exciting circuits. Taking the ferromagnetic particle as an example, the inductance-change of a coil caused by a ferromagnetic particle with a radius of $r_{a}$ can be expressed as (7) [23]:

(7) $$\Delta L=\frac{(\sqrt{5}-1)\mu_{0}\mu_{r}N^{2}r_{a}^{3}}{l^{2}}.$$

Here, $\mu_{0}=4\pi \times 10^{-7}H/m$ is the permeability of the vacuum, $\mu_{r}$ is the relative permeability, N is the number of turns of the coil, and l is the width of the coil.

The impedance differences between the exciting circuits of the two sensors, as shown in Figure 2a,b, are given by:

(8) $$\begin{array}{l} \Delta Z_{a}=j\omega \Delta L\\ \Delta Z_{b}=\frac{j\omega \Delta L}{(1-\omega^{2}C(L+\Delta L)+iCr\omega )(1-\omega^{2}CL+iCr\omega )} \end{array}$$

**Figure 3:**
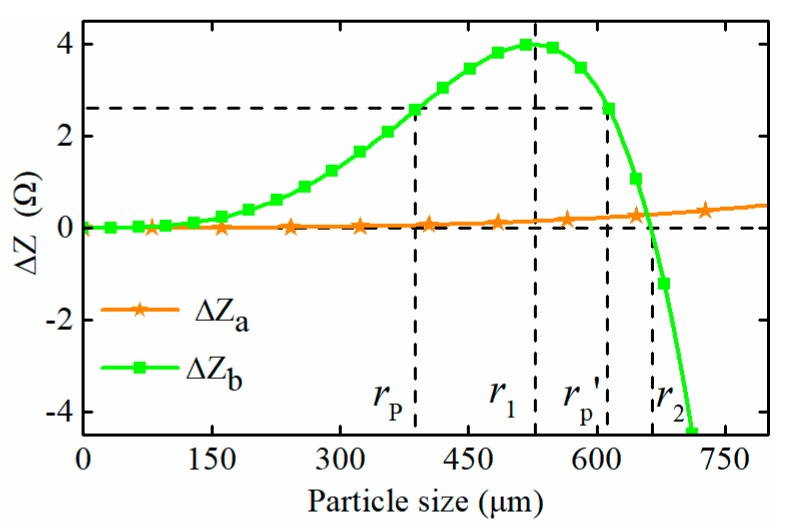
The impedance difference between exciting circuits of different sensors.

To characterize the sensitivity of the two sensors, the impedance differences between the exciting circuits of each sensor are calculated by MATLAB (MathWorks, USA) and shown in Figure 3. During the calculation, the equivalent inductance of the exciting coils is $L_{q1}=L_{q2}=270.2\;\mu H$, which is obtained from experimental measurement, the exciting frequency is set to $f_{0}\;=134.5\;\mathrm{kHz}$, and the corresponding resonant capacitances are $C_{1}=C_{2}=5.17\;\mathrm{nF}$. It can be seen that for the sensor with a non-resonance principle, the impedance difference slowly grows with the increase of the particle diameter, and that it is merely 0.41 Ω when the diameter of the ferromagnetic particle is 750 μm. However, for the sensors with resonant exciting coils, the impedance difference rises rapidly with the increase of particle diameter, reaches a peak value (3.99 Ω) at the position of r1 (528 μm), and then decreases sharply. Therefore, the obvious impedance difference between the exciting circuits of the proposed sensor signifies that the parallel resonant exciting coil does improve the sensitivity of the sensor to a certain extent. However, the nonlinear characteristics of the impedance difference mean that different sized particles, such as the particles with the diameter of $r_{p}$ and $r_{p}\prime$, may lead to the same impedance change, and even the impedance change, caused by the particle larger than $r_{2}$ in diameter, turns negative, which means that the large ferromagnetic particle may be recognized as a non-ferromagnetic particle. Therefore, for correctness of the particle detection result, the effective detection range of the proposed sensor is restricted to (0,$r_{1}$).

**Figure 4:**
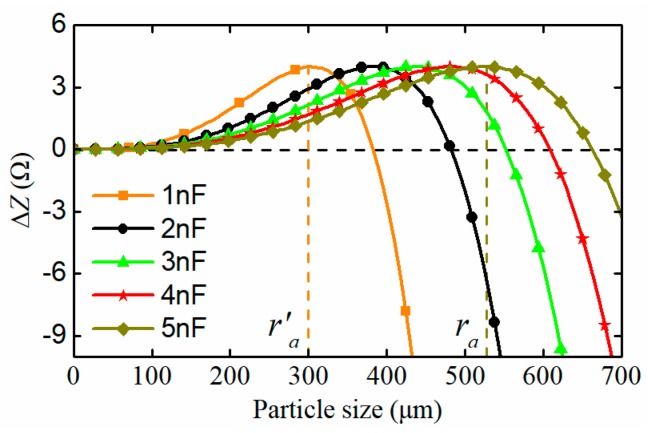
Impedance difference between exciting circuits of the proposed sensor with different resonant capacitors.

To effectively monitor the initial abnormal wear stage of the machinery, some measures must be taken to improve the detectability for micro particles. It is calculated that for the proposed sensor, the resonance capacitance (or resonance frequency) greatly affects the peak position of $\Delta Z_{b}$. The impedance differences between the two exciting circuits with different resonance capacitors are displayed in Figure 4. It can be seen that with the decrease of the capacitance, the impedance difference curve shifts to the left, which reduces the particle detection range of the sensor to $\left(0,r_{a}{}^{\prime }\right)$, but enhances the impedance difference between the two exciting circuits caused by micro particles. Therefore, the smaller resonance capacitance (higher resonance frequency) contributes to the detection of micro wear particles. However, that greatly increases the current through the exciting coils and makes the sensor produce more heat, which is harmful to the reliability of the sensor. Meanwhile, the excessive field frequency increases the magnetic losses in particles, which weakens the detectability for ferromagnetic particles. Considering the above factors, a real well-selecting experiment was conducted, and the results showed that a resonant capacitance of 1nF is appropriate for the detection of ferromagnetic particles. In this situation, the detection range of the sensor was restricted to (0, 300) μm.

The impedance change of exciting coils caused by particles leads to current redistribution, which is one of the key factors of improving the sensitivity of the sensor. Under this circumstance, the current difference between exciting coils, for the sensors shown in Figure 2a,b, can be expressed as (9) and (10), respectively:

(9) $$\Delta I_{a}=I_{0}\left(\frac{1+\Delta Z_{a}/Z_{a}}{2+\Delta Z_{a}/Z_{a}}\right)\left(1-\frac{1}{1+\Delta Z_{a}/Z_{a}}\right)$$

(10) $$\Delta I_{b}=I_{0}\frac{Z_{b}}{Z_{a}}\left(\frac{1+\Delta Z_{b}/Z_{b}}{2+\Delta Z_{b}/Z_{b}}\right)\left(1-\frac{1}{1+\Delta Z_{a}/Z_{a}}\right).$$

Note here that, when the particle diameter is distributed in the range $\left(0,r_{a}{}^{\prime }\right)$, $Z_{b}>Z_{a}$ and $\Delta Z_{b}/Z_{b}>\Delta Z_{a}/Z_{a}$. Therefore, we obtain:

(11) $$\Delta I_{b}=\frac{Z_{b}}{Z_{a}}\left(\frac{1+\Delta Z_{b}/Z_{b}}{2+\Delta Z_{b}/Z_{b}}\right)\left(\frac{2+\Delta Z_{a}/Z_{a}}{1+\Delta Z_{a}/Z_{a}}\right)\Delta I_{a}\gg \Delta I_{a}.\;$$

**Figure 5:**
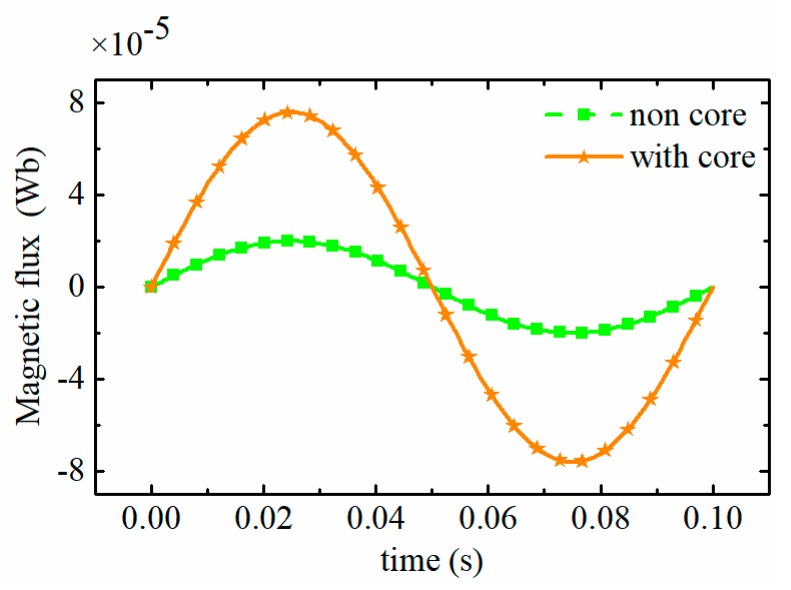
Magnetic flux of the inductive coil.

The combination of (3) and (11) implies that the parallel resonant exciting coil can essentially improve the induced electromotive force. Meanwhile, Equations (2) and (3) indicate that increasing the magnetic flux through the inductive coil is helpful to further enhance the detectability for micro wear particles and boost the sensitivity of the sensor. Therefore, an amorphous iron core is added to the inductive coil. For the inductive coil, the difference in the magnetic flux density between the two exciting coils can be equivalent to a weak external magnetic field $H_{p}$, which produces the magnetic flux of the inductive coil. Based on the equation of B=μH,φ=∑∫Bds, it can be obtained that a ferrite core with a high permeability can boost the external magnetic field and enhance the magnetic flux of the inductive coil. To demonstrate the enhancement effect of the magnetic flux by the amorphous core, a simulation was conducted using the software of COMSOL Multiphysics (COMSOL, Stockholm, Sweden). The simulation parameters used were obtained from the experimental system (illustrated in Section 3). The magnetic fluxes of the inductive coil caused by a 100 μm iron particle for the sensors are displayed in Figure 5. It can be seen that the magnetic flux through the inductive coil of the sensor with the amorphous core increases significantly. In this case, a larger induced electromotive force is produced by the inductive coil.

To further magnify the induced electromotive force caused by particles, the series resonance principle is adopted for the inductive coil and the capacitor *C*_3_ also needs to meet the resonance condition as (4). It is noteworthy that the resonance frequency should maintain a consistent value with the exciting frequency $f_{0}$ and the inductive coil can be regarded as a power source. Under the series resonant state, the current through the coil reaches a peak as (12), and the output signal of the sensor can be expressed as (13). The result shows that the series resonant inductive coil magnifies the output signal of the sensor, and the magnification can be comprehensively described as the quality factor of the induction coil. In this situation, the stray capacitance of the coil and the equivalent series resistance of the resonant capacitor cannot be neglected, so it is difficult to directly calculate the quality factor. We measured the quality factor using a digital electric bridge tester (TH2821B) and obtained an approximate value of 3.22, which indicates that the output signal of sensor $E_{s}\approx 3.22E_{0}$:

(12) $$I_{3}=\frac{E_{0}}{\left(r_{3}+j\omega L_{3}+1/j\omega C_{3}\right)}\approx \frac{E_{0}}{r_{3}}$$

(13) $$E_{s}=I_{3}(r_{3}+j\omega L_{3})=E_{0}\sqrt{1+{\left(\omega L_{3}/r_{3}\right)}^{2}}>E_{0}.$$

Here, $I_{3}$ is the current through the inductive coil under the resonant state, and $E_{0}$ and $E_{s}$ are the induced electromotive forces output by the inductive coil and the sensor, respectively.

Consequently, adding an amorphous iron core to the inductive coil and making it work in the series resonance state are two significant methods of further improving the sensitivity of the sensor.

### 2.3. Particle Signal Measurement Setup

For the proposed sensor, because of the weak inhomogeneity of the magnetic field between the exciting coils, the initially induced electromotive force interference is produced when no particles pass through the sensor. By analyzing the characteristics of the sensor signal, it can be obtained that the real output signal is composed of the effective particle signal, initially induced electromotive force interference, and environmental interference. The real sensor signal can be expressed as:

(14) $$E_{s}=E_{0}\sqrt{1+{\left(\omega L_{3}/r_{3}\right)}^{2}}=(E(r_{a},v)\sin(\omega_{1}t+\varphi_{2})+E(\Delta ))\sin(\omega_{0}t+\varphi_{1})+N(t)$$

where, $E(r_{a},v)\sin(\omega_{1}t+\varphi_{2})$ is the effective particle signal, $E(\Delta )\sin(\omega_{0}t+\varphi_{1})$ is the initially induced electromotive force interference, $\omega_{0}$ and $\omega_{1}$ are the angular frequencies of the exciting signal of the sensor and the effective particle signal respectively, and N(t) is the Gaussian noise resulting from environmental interference.

**Figure 6:**
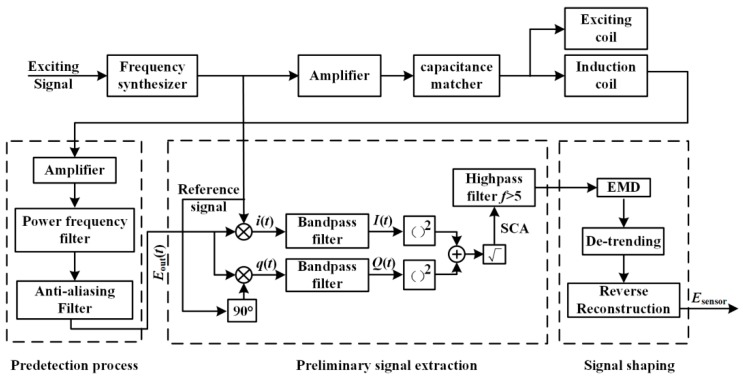
Block diagram of the signal measurement system.

A measurement system for weak signals is crucial for the detection of wear particles. For satisfying the high real-time requirements of online wear monitoring, a new signal extraction method, based on a modified lock-in amplifier (MLIA) and empirical mode decomposition (EMD), is proposed. Compared with conventional peak-detection (PD) algorithms [17,18,20], the proposed method is much simpler and faster. It can adapt to circumstances with an extremely low signal-to-noise ratio (SNR). Figure 6 shows the block diagram of the signal measurement system. The frequency synthesizer is used to adjust the frequency of the exciting signal to satisfy various monitoring situations. A capacitance matcher is applied to match suitable capacitances for sensor coils. The process of particle signal extraction includes the pre-detection process, preliminary signal extraction, and signal shaping. In the pre-detection process, the raw signal of the sensor is amplified and then filtered by a power frequency filter and an anti-aliasing filter to remove the 50 Hz interference and the high-frequency interference which is generally caused by mechanical vibration of the sensor. For preliminary signal extraction, a modified lock-in amplifier (MLIA) is proposed. In contrast to a conventional lock-in amplifier (LIA), the MLIA adopts two Bessel-type band-pass filters with a center frequency $f_{0}$ due to the essential feature of the sensor signal, and the effective particle signal is amplitude-modulated by a sinusoidal signal with a frequency of $f_{0}$. Besides that, to quickly eliminate the initially induced electromotive force interference, a Bessel high-pass filter with a cut-off frequency of 5 Hz was used. Because the extraction effect of the particle signal is relevant to the function of these filters and SNR of the raw signal, to adapt the detection requirement of the particles with different speeds, the raw signal is always under-filtered by these filters. Therefore, some unfiltered Gaussian interference still exists in the particle signal, which lowers the detection effect for particles, especially for particles with a low speed. Hence, the particle signal-shaping method based on the EMD is proposed.

In the procedure for preliminary signal extraction, the reference signal of MLIA is set to $A\sin(\omega_{0}t+\varphi_{3})$, which has the same frequency as the exciting signal. After that, the raw signal is multiplied by both the reference signal and a signal in quadrature with respect to a reference signal of $A\cos(\omega_{0}t+\varphi_{3})$. The signals of i(t) and q(t) can be obtained as (15) and (16), respectively. It can be seen that i(t) and q(t) consist of three parts: the amplitude component, high-frequency part (frequency is $2f_{0}$), and noise sector:

(15) $$\begin{array}{l} i(t)=((E(r_{a},v)*\sin(2\pi f_{1}+\varphi_{2})+E(\Delta ))*\sin(2\pi f_{0}t+\varphi_{1})+N(t))A\sin(2\pi f_{0}t+\varphi_{3})\\ =\frac{A}{2}(E(r_{a},v)*\sin(2\pi f_{1}+\varphi_{2})+E(\Delta ))*\cos(\varphi_{1}-\varphi_{3})-\frac{A}{2}(E(r_{a},v)*\sin(2\pi f_{1}+\varphi_{2})\\ +E(\Delta ))*\cos(2*2\pi f_{0}t+\varphi_{1}+\varphi_{3})+N(t)*A\sin(2\pi f_{0}t+\varphi_{3}) \end{array}$$

(16) $$\begin{array}{l} q(t)=((E(r_{a},v)*\sin(2\pi f_{1}+\varphi_{2})+E(\Delta ))*\sin(2\pi f_{0}t+\varphi_{1})+V(t)+N(t))*A\cos(2\pi f_{0}t+\varphi_{2})\\ =\frac{A}{2}(E(r_{a},v)*\sin(2\pi f_{1}+\varphi_{2})+E(\Delta ))*\sin(\varphi_{1}-\varphi_{3})+\frac{A}{2}(E(r_{a},v)*\sin(2\pi f_{1}+\varphi_{2})\\ +E(\Delta ))*\sin(2*2\pi f_{0}t+\varphi_{1}+\varphi_{3})+N(t)*A\cos(2\pi f_{0}t+\varphi_{3}) \end{array}$$

After the MLIA’s band-pass filters, the high-frequency component and most of the noise interference can be removed. Therefore, the following signals are obtained:

(17) $$I(t)=\frac{A}{2}(E(r_{a},v)*\sin(2\pi f_{1}+\varphi_{2})+E(\Delta ))*\cos(\varphi_{1}-\varphi_{3})$$

(18) $$Q(t)=\frac{A}{2}(E(r_{a},v)*\sin(2\pi f_{1}+\varphi_{2})+E(\Delta ))*\sin(\varphi_{1}-\varphi_{3}).$$

The estimation of the specific component amplitude (SCA) is given by (19). There are two sectors in the SCA: a sinusoidal component with a frequency of $f_{1}$, which involves the effective particle signal, and a direct component that reflects the amplitude of the initially induced electromotive force interference. Therefore, a Bessel high-pass filter with a cut-off frequency of 5 Hz is used to remove the DC interference component, and the effective particle signal is then obtained as (20):

(19) $$\mathrm{SCA}=\sqrt{I{\left(t\right)}^{2}+Q{\left(t\right)}^{2}}=\frac{A}{2}(E(r_{a},v)*\sin(2\pi f_{1}+\varphi_{2})+E(\Delta ))$$

(20) $$E_{\mathrm{sig}}=\frac{A}{2}(E(r_{a},v)*\sin(2\pi f_{1}+\varphi_{2}).$$

That the cut-off frequency of the high-pass filter is 5 Hz means that the allowable minimal speed of particles passing through the sensor is $v=f_{1}*l=5*11\;\times \;10^{-3}=5.5\;\times \;10^{-2}\;m/s$, and the corresponding allowable minimum quantity of flow is $V=\pi vd^{2}/4=0.127\;L/\min$. Here, l is the outer distance between the exciting coils and d is the inner diameter of the sensor.

Although the modified lock-in amplifier can preliminarily extract the weak particle signal and greatly improve the SNR of the signal, there is still some unfiltered Gaussian interference which influences the accurate judgment of the signal amplitude. Therefore, the signal-shaping method based on the EMD-RRC (empirical mode decomposition and reverse reconstruction) is adopted. EMD is an adaptive time-frequency signal processing method used to decompose non-stationary or nonlinear data into several elementary intrinsic mode functions (IMFs), which contain the local features of the raw signal at different time scales. The detailed decomposition process is stated in [24,25]. The preliminarily extracted particle signal can be decomposed by the EMD method as:

(21) $$E_{\mathrm{sig}}=\sum_{i=1}^{k}c_{i}(t)+r(t)$$

where, $c_{i}(t)$ is the *i*th intrinsic mode function and r(t) is the residual term.

Based on the theory of the EMD, the low-order IMFs contain the high-frequency component of the raw signal, and the high-order IMFs and the residual term represent the low-frequency trend component of the signal. Considering the preliminarily extracted particle signal, in order to eliminate the residual interference, the trend component with a low frequency should be removed first. Hence, a trend component identification method is adopted. In this method, the trend component is identified as [10]:

(22) $$m(t)=\sum_{i=k_{1}}^{k}c_{i}(t)+r(t)\;$$

where, $k_{1}$ is the trend order of IMFs which satisfies:

(23) $$\begin{array}{l} \prod_{i=k_{1}}^{k}\left(|\mathrm{Mean}(c_{i}(t))|-H_{T}\right)>0\\ \prod_{i=1}^{k_{1}-1}\left(|\mathrm{Mean}(c_{i}(t))|-H_{T}\right)<0 \end{array}$$

where, Mean(.) denotes the mean function, and $H_{T}=0.05|\mathrm{Mean}(r(t))|$ is the threshold.

To further eliminate the high-frequency interference, a reverse reconstruction method is proposed to reconstruct the signal of the particle. This method gradually adds lower-order IMFs to the detrended highest-order IMF, which produces a series of reconstruction signals expressed as:

(24) $$E_{\mathrm{rsig}}^{j}=\sum_{i=k_{1}-j}^{k_{1}-1}c_{i}(t).$$

The best denoising effect means the maximal correlation between the particle signal and an ideal sinusoidal signal. Hence, the synthesized correlation coefficient as (25) is used to evaluate these reconstructed signals and to select the best reconstruction order:

(25) $$\rho_{\mathrm{rsig}}^{j}=\frac{\mathrm{COV}(E_{\mathrm{rsig}}^{j},E_{\mathrm{std}})}{\sqrt{E_{\mathrm{rsig}}^{j}}\sqrt{E_{\mathrm{std}}}}.$$

Here, COV(.) denotes the covariance function and $E_{\mathrm{std}}$ is an ideal sinusoidal signal.

The array of synthesized correlation coefficients for the different reconstruction particle signals is established as:

(26) $$\rho_{\max}=\max(|\rho_{\mathrm{rsig}}^{1}|,|\rho_{\mathrm{rsig}}^{2}|,\ldots ,|\rho_{\mathrm{rsig}}^{j}|).$$

Combining Equations (24)–(26), the best reconstruction signal is expressed as:

(27) $$E_{\mathrm{out}}=\sum E_{\mathrm{rsig}}^{j}*(\mathrm{sgn}(|\rho_{\mathrm{rsig}}^{j}|-\rho_{\max})+1).$$

The signal extraction process is simulated by MATLAB SIMULINK and the signal-to-noise ratio (SNR), as shown Equation (28), is used to evaluate the effect of the proposed signal measurement system. In addition, to illustrate the influence on the signal detection effect by the initially induced electromotive force interference, the signal-to-harmonics ratio (SHR) is defined as (29).

(28) $$\mathrm{SNR}=10\log_{10}{}^{\left(P_{p}/P_{N}\right)}$$

(29) $$\mathrm{SHR}=\frac{E_{p}{|}_{p-p}}{E_{0}{|}_{p-p}}.$$

Here, $P_{p}$ and $P_{N}$ are the power of the effective particle signal and the noise signal respectively, $E_{p}$ is the effective particle signal, $E_{0}$ is the initially induced electromotive force, and the subscript p-p means the peak-to-peak value.

**Figure 7:**
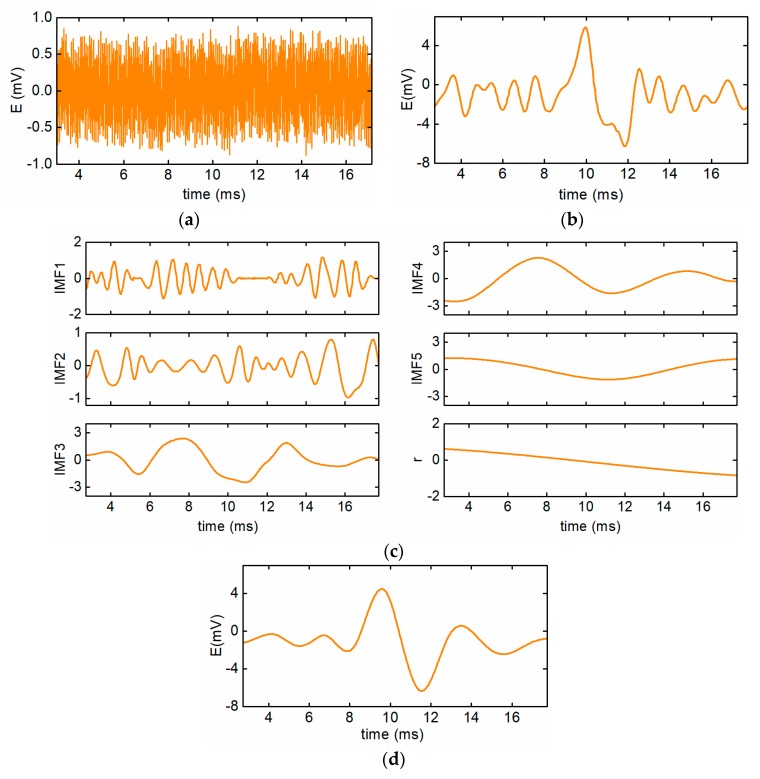
The simulation of the signal extraction process. (**a**) The raw signal of the sensor, (**b**) the preliminarily extracted particle signal, (**c**) the decomposed particle signal, and (**d**) the shaped particle signal.

The simulation is conducted on the condition that the effective particle signal is $E_{0}=5\times 10^{-5}\sin(2\pi f_{0}t)$, SHR equals 1/100, the variance of Gaussian noise is 1e-8, and the signal amplification factor is 100. In this situation, the raw signal of the sensor is demonstrated in Figure 7a, which shows that the particle signal is fully submerged in the interference, and the SNR of the raw signal is as low as –21.37 dB. The preliminarily extracted particle signal is displayed in Figure 7b. It can be seen that the interference component is greatly removed from the raw signal, however, the residual interference still influences the amplitude recognition. In the process of signal-shaping, the preliminarily extracted signal is decomposed into several IMFs and a residual component by the EMD method, as shown in Figure 7c. Based on Equations (21)–(25), the IMF5 and the residual component are regarded as low-frequency trend components and the IMF1 and IMF2 are treated as high-frequency interference. After eliminating all the interference, the reconstructed signal can be obtained, as shown in Figure 7d. It shows that the shaped particle signal has obvious sinusoidal characteristics.

To evaluate the validity of the proposed signal extraction and shaping method, the SNR values of the raw signal, preliminarily extracted signal, and shaped signal are calculated and presented in Table 1. The result illustrates that the SNR of the signal is greatly improved, which contributes to boosting the particle detection effect of the sensor.

### 2.4. Analysis of the Computational Cost and Performance of Methods

As wear particles are monitored in real time by an electromagnetic wear particle detector, the computational efficiency of particle signal extraction algorithms and the correctness of detection results are of important concern. Therefore, in this section, a comparative analysis, involving the computational cost and extraction effect of particle signals incurred by the application of RSD-FC (resonance-based signal decomposition method and fractional calculus) [20], VMD-based method (variational mode decomposition) [26,27,28], and EMD-RRC (empirical mode decomposition and reverse reconstruction), is presented.

With respect to EMD and VMD, the algorithms decompose raw signals into several sub-signals (modes). However, the implementation of VMD requires first performing a Hilbert transform which involves an EMD process, so the VMD carries on a computational cost higher than the EMD. Besides that, the VMD requires a predetermined number of decomposition level *k*, which greatly influences its decomposition effect and computational efficiency [28]. Moreover, it’s difficult to adjust the value of *k* for the optimal decomposition effect self-adaptively. The RSD-FC expresses a signal as the sum of a ‘high-resonance’ component which generally represents the interferences and a ‘low-resonance’ component which characterizes the particle signal. To achieve this goal, a morphology component analysis needs to be conducted, in which, an iterative optimization algorithm is utilized to update the transform coefficient matrices [20], so the method requires extensive calculations. To evaluate the computational efficiency, the preliminarily extracted particle signal with a sampling time of 1 s, extended from the data of Figure 7b, is processed using different algorithms running on a PC (Intel(R) Core(TM) i7-4720HQ CPU, 2.60 GHz, 8 GB RAM, Windows 10 operating system). For effective detection of wear particles with high speed, the sampling frequency is set to 3000 Hz. The theoretical peak-to-peak value of the particle signal output by the sensor is 10 mV. The performance of the algorithms is evaluated using the mean signal-to-noise ratio (MSNR), mean peak-to-peak value (MPPV), and mean relative amplitude error (MRAE):

(30) $$MRAE=\frac{1}{n}\sum_{i=1}^{n}\left|\frac{T_{i}-M_{i}}{T_{i}}\right|\times 100\%$$

where, $T_{i}$ and $M_{i}$ represent respectively, the theoretical and measured peak-to-peak value of particle signals, and *n* is the number of samples.

**Figure 8:**
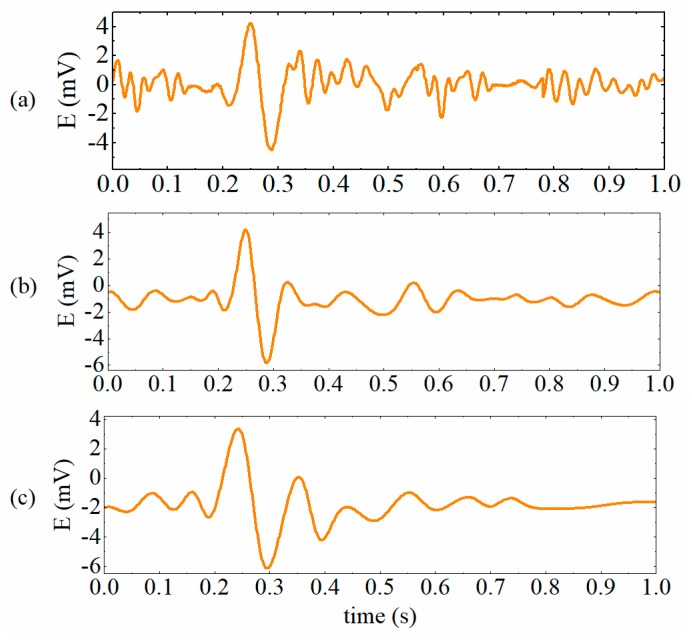
The extraction results of different algorithms applied on the preliminarily extracted signals. (**a**) resonance-based signal decomposition method and fractional calculus (RSD-FC), (**b**) variational mode decomposition (VMD)-based method with k = 7, (**c**) empirical mode decomposition and reverse reconstruction (EMD-RCC).

The extraction results of particle signals by RSD-FC, VDM-based method (*k* = 7), and the EMD-RRC are demonstrated in Figure 8a–c, which shows that the residual interferences in preliminarily extracted particle signals are removed to different degrees. The computational time and the performance of the algorithms are displayed in Table 2. It can be seen that all the methods do improve the SNR of signals to a certain degree and the MSNR of the extracted particle signals are higher than 10, which contributes to the effective detection of micro-particles. Furthermore, among these methods, the computational time of the RSD-FC is the longest and reaches to 1.9548 s, which is much larger than the sampling time (1 s). Therefore, it is difficult to guarantee real-time performance of particle detection sensors. Besides that, the correctness of the particle detection results is relatively poor. The MPPV and MRAE of particle signals extracted by the RSD-FC are 9.26 mV and 7.4%, respectively. For the VMD-based method, with the increase of the number of decomposition level *k*, the computational time rises accordingly. Moreover, comprehensively considering the evaluation indicators, the VMD-based method with k = 7 performs best (MSNR = 13.357 dB, MPPV = 9.71 mV, and MRAE = 2.9%). However, in this case, the computational time is 1.4942 s, which is also larger than the sampling time (1 s). While for the proposed EMD-RRC method, the MPPV and the MRAE of signals are 9.68 mV and 3.2%, respectively. Although, they are slightly lower than that of the VMD-based method with k = 7, the average computational time is only about 0.83 s which is sufficient to process the data of 1 s long with 3000 samples in real time. In summary, the proposed method is sufficiently fast for on-line application in terms of both computational efficiency and detection quality.

## 3. Experiment

### 3.1. Experimental System

**Figure 9:**
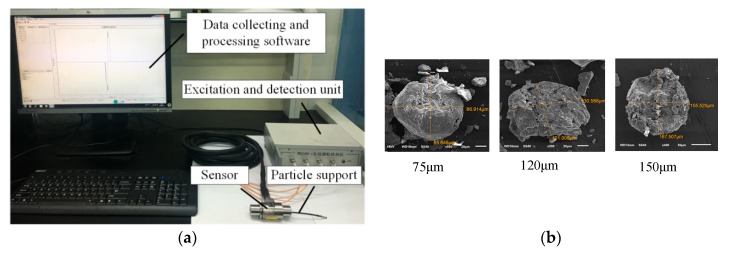
Experimental system. (**a**) The particle detection system, (**b**) the selected iron particles.

To verify the improvement of the sensitivity and the detectability of the sensor contributed by the resonance mechanism, the amorphous iron core, and the proposed signal measurement system, the detection efficiencies of the conventional and proposed sensors for wear particles were tested. The complete experimental system, as shown in Figure 9a, consists of the sensor, the excitation and detection unit, which is used to supply the exciting signal and to extract the particle signal, and the data collecting and processing software. The core parameters of the sensors adopted in the experiments are listed in Table 3. Furthermore, some sphere-like iron particles with the diameters of 75, 120, and 150 μm are selected by the scanning electron microscope as target particles, as shown in Figure 9b. The previous experimental research shows [29] that the lubricating oil does not affect the signal of the sensor, so the sensitivity analysis experiments are conducted under an oil-less condition.

**Figure 10:**
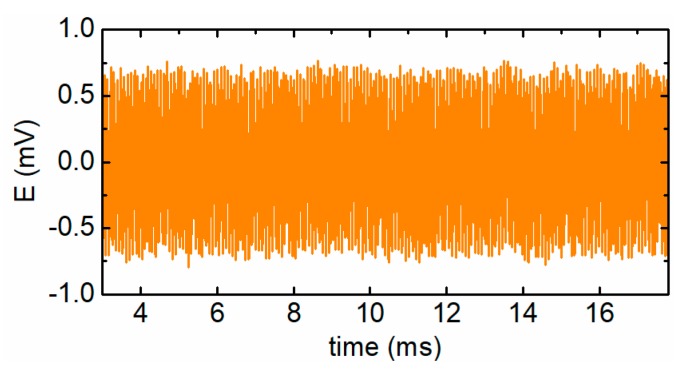
The raw signal of the proposed sensor.

**Figure 11:**
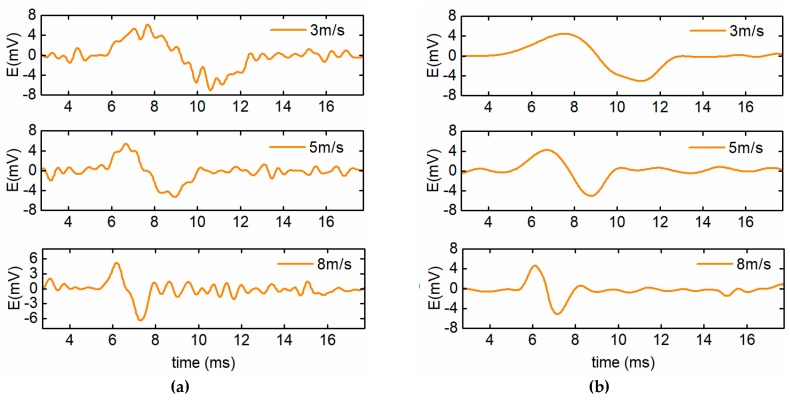
The signal of particles with different speeds. (**a**) The preliminarily extracted particle signals and (**b**) the shaped particle signals.

During the experiment, the measurement data shows that the initially induced electromotive forces of the sensors are about $E_{0}=7.3\times 10^{-4}\sin(2\pi f_{0}t)$ V and the Gaussian noise is very apparent. In this case, the particle signal is totally submerged in the inference. Taking the proposed sensor as an example, Figure 10 shows the raw signal of the sensor caused by a particle with the diameter of 120 μm. Because the particle speed may influence the signal extraction effect to a certain degree, particle detection experiments were conducted when the particle moved at the speed of 3 m/s, 5 m/s, and 8 m/s, respectively. The preliminarily extracted particle signal and the shaped particle signal are shown in Figure 11a,b, respectively. The results indicate that for the preliminarily extracted signals, a better detection is achieved at a higher particle speed. Moreover, after the signal shaping, the residual interference is further removed and the signals of the particle with different speeds can be effectively extracted. The SNR and peak-to-peak values of the particle signals are listed in Table 4, which shows that the proposed particle signal extraction method can greatly enhance the SNR of the particle signals and benefit the detection of micro wear particles. In addition, the peak-to-peak values of the signals are approximately consistent, which means that the signal measurement system has high fidelity.

### 3.2. Sensitivity Comparison for Ferromagnetic Particle Detection

**Figure 12:**
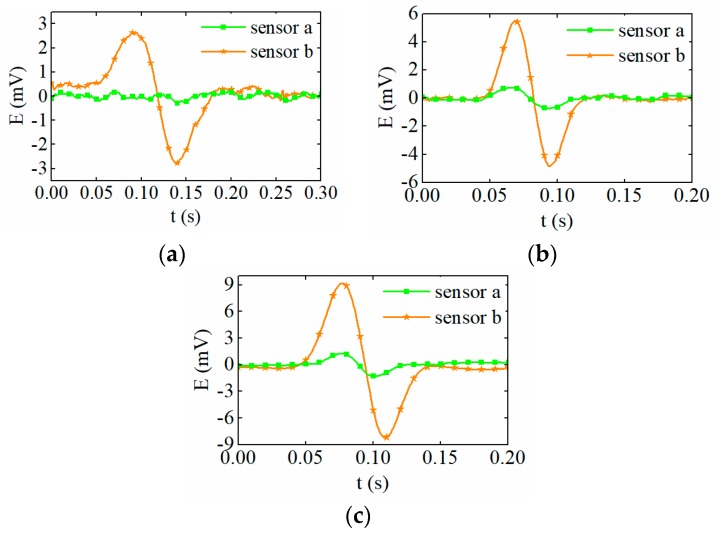
The particle signal output by the sensors. (**a**) 75 μm, (**b**) 120 μm, (**c**) 150 μm.

To illustrate the sensitivity improvement by the proposed methods, both the conventional sensor, as shown in Figure 2a, and the proposed sensor, as shown in Figure 2b, were tested. Figure 12 shows the output signal of the sensors caused by the different sizes of ferromagnetic particles. In the figure, the green curve illustrates the signal output by the conventional sensor, and the orange curve represents the output signal of the proposed sensor, which adopts a resonance principle and an amorphous iron core. It can be seen that, for the conventional sensor, it is difficult to effectively detect iron particles less than 100 μm in diameter and the peak value of the induced electromotive force caused by a 100 μm iron particle is only 0.59 mV. However, for the proposed sensor, the signal amplitude of the particle with the diameter of 75 μm reaches 2.6 mV, which is much greater than that of the conventional sensor.

**Figure 13:**
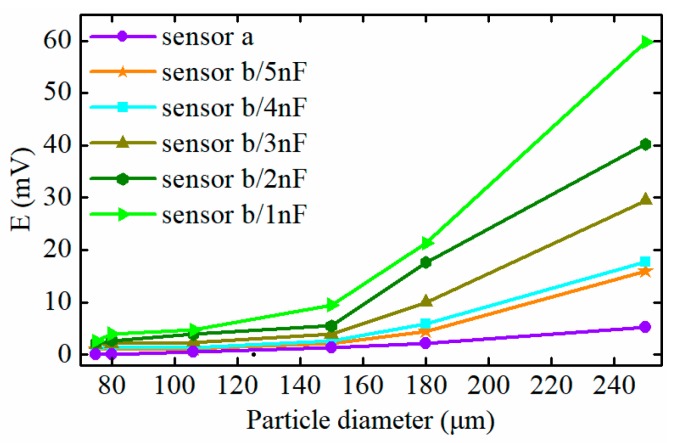
The comparison analysis of sensor sensitivity.

A comparison analysis of the detection result of the conventional sensor and the proposed sensor with various resonant capacitances is presented in Figure 13. It can be seen that the particle signal output by the proposed sensor is much larger than that of the traditional one, and with the decrease of the exciting capacitance, the sensitivity of the sensor gradually increases. The amplitude of the signal caused by a 75 μm iron particle, when the exciting capacitance equals 1 nF, is 2.6 mV, which is much greater than that under the circumstance of $C_{1}=C_{2}=5\;\mathrm{nF}$ (1.06 mV), and the increasing trend tends to be more evident for larger particles. However, excessive reduction of the resonant capacitance leads to a stronger eddy current effect in ferromagnetic particles and increases the current through the exciting coil rapidly, which may weaken the detectability for ferromagnetic particles and greatly reduce the reliability of the sensor. Therefore, a 1 nF resonance capacitance for the exciting coil is finally used for ferromagnetic particle detection.

### 3.3. Wear Monitoring in a Real Oil Environment

**Figure 14:**
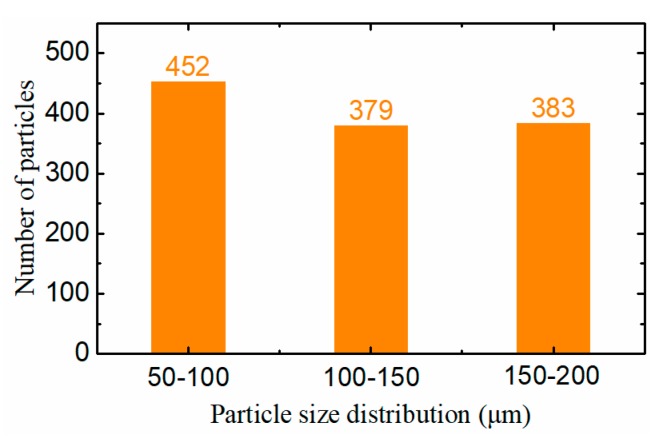
The statistical distribution of the wear particles.

To verify the detection effect of the sensor in a real oil environment, the sensor was assembled in the lubrication system with large ferromagnetic wear particles, comprised of 20 particles with a diameter of 80–100 μm, 20 particles with a diameter of 120–150 μm, and 20 particles with a diameter of 150–180 μm. These particles were added into the oil to simulate a serious wear fault of the mechanical equipment. The lubricating oil, including the wear particles, were driven by a pump and cycles through the sensor 20 times. By monitoring the wear particles using the sensor, the size distribution and the number of wear particles were estimated. The statistical result is displayed in Figure 14, which shows that the number of detected wear particles greater than 100 μm in diameter is approximately consistent with the standard value (400). However, the number of iron particles smaller than 100 μm in diameter is slightly more than the standard value. The possible reason for this phenomenon is that some parts of the larger wear particles may stick to the inner surface of the pipeline or be ground down to smaller particles by the blades of the pump during its running process. Therefore, based on the experimental result in a real oil environment, it can be concluded that the sensor can effectively monitor the quantity of the wear particles with different sizes, which helps to estimate the wear state of the mechanical equipment and to prevent mechanical failure caused by serious wear.

## 4. Conclusions

The electromagnetic particles’ detection sensor is of great importance due to its prospective application in various fields, and the sensitivity and detectability are still major obstacles in the development of wear particle detectors. Therefore, this paper has proposed that the resonance principle, an amorphous iron core, and a new signal measurement system are adopted to comprehensively improve the sensor sensitivity and detectability. Based on the work, the following conclusions are obtained:

(1) For the three-coil wear particle detector, the parallel resonant exciting coil magnifies the impedance difference between exciting circuits caused by particles. Additionally, the amorphous iron core and the series resonant inductive coil increase the magnetic flux through the coil and enhance the induced electromagnetic force of the sensor, which can comprehensively improve the particle signal more than six times compared to the conventional sensor.
(2) Under the resonance state, the nonlinear characteristics of the impedance difference between exciting circuits of the proposed sensor mean that the effective particle detection range of the sensor is restricted to (0,$r_{a}{}^{\prime }$).
(3) Decreasing the resonant capacitance and increasing the exciting frequency can further improve the detection ability for micro-particles, though this reduces the effective particle detection range of sensors.
(4) By comparing different algorithms, the signal measurement system based on the MLIA and EMD-RRC guarantees the real-time ability for online particle detection and can effectively extract the particle signals from the raw signal with an extremely low SNR (≈−20 dB). The experimental results indicate that based on the proposed method of improving the sensitivity and detectability, the large-calibre (7 mm) sensor can effectively monitor the initial abnormal wear of the heavy machines.

## Figures and Tables

**Table 1:** The signal-to-noise ratio (SNR) of signals.

SNR of Raw Signal	SNR of Preliminarily Extracted Signal	SNR of Shaping Signal
–21.37 dB	3.71 dB	13.181 dB

**Table 2:** Computational times and the performance of different algorithms.

Algorithms	MSNR (dB)	MPPV (mV)	MRAE	Mean Computational Times (s)
RSD-FC	8.854	9.26	7.4%	1.9548
VMD-Based (*k* = 4)	10.347	9.27	7.3%	0.9368
VMD-Based (*k* = 5)	11.755	9.39	6.1%	1.1256
VMD-Based (*k* = 6)	12.982	9.54	4.6%	1.3573
VMD-Based (*k* = 7)	13.357	9.69	3.1%	1.4942
VMD-Based (*k* = 8)	12.793	9.60	4.0%	1.5783
VMD-Based (*k* = 9)	12.584	9.52	4.8%	1.7137
EMD-RRC	13.181	9.68	3.2%	0.8314

**Table 3:** The core parameters of the sensors adopted in the experiments.

Parameters	Conventional Sensor	The Proposed Sensor
Inner diameter of the sensor	7 mm	7 mm
Width of the coils	2 mm	2 mm
Inner diameter of exciting coils	9 mm	9 mm
Number of turns of exciting coils *N*_e_	127	127
Number of turns of exciting coils *N*_i_	110	110
Inner diameter of inductive coil	11 mm	11 mm
Inner diameter of amorphous core	-	9 mm
Outer diameter of amorphous core	-	11 mm
Resonant exciting capacitance *C*_1_, *C*_2_	-	1.0 nF
Resonant inductive capacitance *C*_3_	-	0.63 nF

**Table 4:** The signal-to-noise ratio (SNR) of real signals.

Particle Speed (m/s)	SNR (dB)			Peak-To-Peak Value (mV)
	Raw Signal	Preliminarily Extracted Signal	Shaping Signal	
3	–14.318	9.341	12.933	9.62
5	–15.541	10.366	17.609	9.58
8	–19.917	11.625	15.173	9.55
